# Molecular Dynamics Study on Tip-Based Nanomachining: A Review

**DOI:** 10.1186/s11671-020-03419-5

**Published:** 2020-10-15

**Authors:** Zihan Li, Yongda Yan, Jiqiang Wang, Yanquan Geng

**Affiliations:** 1 Center for Precision Engineering, Harbin Institute of Technology, Harbin, 150001 Heilongjiang People’s Republic of China; 2 The State Key Laboratory of Robotics and Systems, Robotics Institute, Harbin Institute of Technology, Harbin, 150080 Heilongjiang People’s Republic of China

**Keywords:** Tip-based nanomachining, Molecular dynamics simulation, Simulation model, Machining mechanism analysis, Nanometric cutting

## Abstract

Tip-based nanomachining (TBN) approaches has proven to be a powerful and feasible technique for fabrication of microstructures. The molecular dynamics (MD) simulation has been widely applied in TBN approach to explore the mechanism which could not be fully revealed by experiments. This paper reviews the recent scientific progress in MD simulation of TBN approach. The establishing methods of the simulation model for various materials are first presented. Then, the analysis of the machining mechanism for TBN approach is discussed, including cutting force analysis, the analysis of material removal, and the defects analysis in subsurface. Finally, current shortcomings and future prospects of the TBN method in MD simulations are given. It is hopeful that this review can provide certain reference for the follow-up research.

## Introduction

Micro/nanomanufacturing technology has been widely used in various areas, including environment, energy, biology, medicine, national defense, and other fields, which plays an increasing important role in promoting national development and social progress [1–4]. To realize high-precision micro/nanofabrication, the atomic force microscopy (AFM) probe-based mechanical machining approach known as TBN has simple equipment and operations, nanoscale machining accuracy with low environmental requirement, and it has been shown to be a powerful and feasible approach to fabricate microstructures [5, 6]. Until now, nanodots, lines/grooves, two-dimensional (2D)/three-dimensional (3D) structures, and even nanostructures on curved surfaces known as the major components of nanodevices have already been successfully fabricated by TBN method [7].

To fabricate nanostructure with higher precision, a deep understanding in the machining process of TBN methods is needed. Many scholars have investigated the mechanism of TBN approach with experiments in various materials, such as metals [8], semiconductors [9, 10], and polymers [11]. However, it is difficult to further investigate the inner mechanism of TBN approaches by experiments. The molecular dynamics (MD) simulation has been demonstrated to be a powerful tool to explore the nanomachining process which could not be fully revealed by experiments [12, 13]. Compared with the experimental method, the MD method can better explain the microscale mechanism of material removal and surface generation through the analysis of cutting forces, stress state, energy dissipation, and surface topography [12].

In the past, some scholars have already used MD simulation technology to investigate the TBN processes. Fang et al. investigated the effects of the scribing feed and cone angle on the nanolithography process [14, 15]. Isono and Tanaka analyzed the effects of temperature, machinability and interatomic forces of the nickel metal [16, 17]. Yan et al. studied the tip geometry effects in AFM-based lithography process [18]. Currently, some novel TBN processes have been investigated with MD simulations. Xiao et al. compared the difference between static ploughing method and dynamic ploughing method, and found smaller nanostructures could be fabricated with dynamic ploughing method [19]. Geng et al. have performed MD simulations of load-controlled nanoscratching by directly applying a constant normal load on the probe [20]. In order to fabricate nanogrooves with higher density, the minimum feed (MF) should be studied. Ren et al. presented a novel approach which involves a coarse-to-fine criterion to determine MF with the use of MD simulations [21]. The results show that MF of high accuracy is obtained. However, in the previous review paper [12, 13, 22–24], these new progresses of the MD simulation technology used for TBN process are not included. Thus, in this review, we focus on the recent state-of-the-art of the MD simulation for TBN methods. The modeling technologies in various materials and novel machining methods are first discussed. Then, the mechanism of TBN methods is reviewed, including cutting force analysis, the analysis of material removal, and the defects analysis in subsurface. Finally, the remaining challenges and future prospect in MD simulation of TBN is also given in this review.

## Simulation Methods

To obtain accurate prediction results, it is necessary to optimize the simulation model and related processing configuration. The simulation model mainly contains atomic configuration and potential function. The atomic configuration can be categorized into crystalline and amorphous structure. The atoms in crystalline materials such as copper, iron, silicon, etc. are arranged in a regular and orderly manner and the amorphous materials like polymers are composed of the irregular molecular chains. Material behavior in nanoscale is represented in MD simulations by means of potential functions and various potential functions have been proposed and utilized to simulate material behavior in atomistic simulations. Besides, the processing configuration should also be considered, such as the various machining conditions and machining approaches. The following sections will present the methodologies of model establishment and the processing configuration.

### Establishment of MD Model

**Fig.1:**
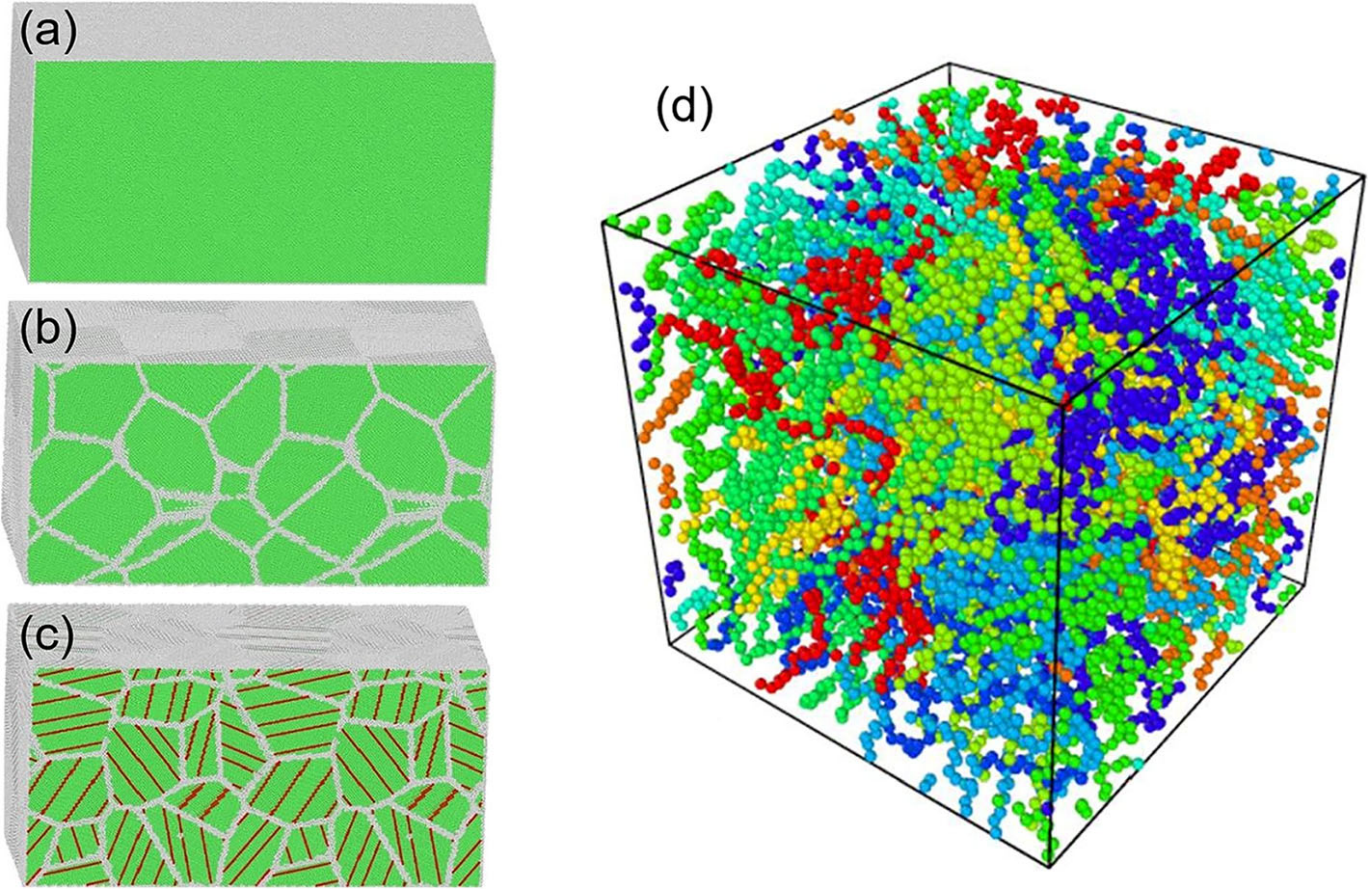
**a** Microstructure of single crystal Cu. **b** Microstructure of nanopolycrystalline. **c** Microstructure of NT polycrystalline. Atoms are colored according to CNA values, as green and white stand for FCC and defect atoms [25]. **d** The equilibrated united-atom model of amorphous PE, the model was colored by different molecular chains [26]

The internal atomic configuration of crystalline materials are various, such as copper (face-centered cubic), iron (body-centered cubic), titanium (close-packed hexagonal), and silicon (diamond structure) [27, 28]. Most of crystalline materials are in the form of single crystalline and polycrystalline structure. The single crystal solid has an atomic structure that repeats periodically across its whole volume with the absence of defects. Duplicating the unit cell in all three spatial direction can easily establish the single crystal model [13]. When establishing atomic MD model, the various surface orientation should be considered, which can influence the machining properties of workpiece [29–31]. The polycrystalline structure has the structure with different grain sizes, which was constructed following the Voronoi tessellation method [32]. Nanotwined (NT) material is a kind of special structures of polycrystalline which has become more important research objects due to its outstanding mechanical properties, such as ultra-high strength, good ductility, and high fracture toughness [33–36]. In this review, the method to construct NT polycrystalline Cu is given as an example [25]: first, a multilayer that consists of multiple single crystal Cu layers of equal thickness is built and TB forms between adjacent layer. Second, the angle of each grain is calculated. Finally, NT polycrystalline with periodic boundary conditions is generated using Voronoi construction, which accommodates the multilayer and the obtained grain angle. Figure 1a presents the atomic configurations of copper including sing crystalline Cu, polycrystalline Cu, and NT polycrystalline Cu, in which atoms are colored by the common neighbor.

In previous studies, the polymer was modeled with a generic coarse-grained model [37, 38]. For example, the details to establish the model of polyethylene (PE) are given as follows [39]: (i) the initial PE system with 10 molecular chains was obtained with the use of Monte Carlo self-avoiding random walk algorithm [39]; (ii) the first atom of each chain was inserted in an available site of the lattice at first and then the molecular chain begun to grow along a certain direction based on a probabilistic stepwise manner according to the bond length and the unoccupied sites in the cell.; (iii) once the initial density is given, the size of the simulation box is determined. In our simulation, the dimension of the initial simulation box was about 80.06 × 80.06 × 80.06 Å^3^ for the system consisting of 10 PE chains. The MD model of PE is shown in Fig. 1d.

Tip geometry is generally complex and variable including cone shape [40], triangular pyramid [18] and hemisphere (blunt shape) [21], which plays an important role in TBN process. In particular, the shape of the pyramid tool in MD model is consistent with that in the AFM machining process and the spherical tool is consistent with the abrasive grain in the grinding process; thus, the machining mechanism can be better explained by comparing the simulation results with the experimental results. In most of cases, the tip is considered infinitely rigid, which is achieved by retaining the relative positions of the atoms constant and travelling at constant speed [41].

It is also crucial to select the suitable potential energy functions which determine the credibility of the simulation results [21]. EAM is a multibody potential suited for metallic systems [24]. It provides a more realistic description of the metallic cohesion and avoids ambiguity inherited by the volume dependency, which is employed to describe the interaction between metallic atoms, such as copper [42] and iron [43]. The Tersoff [44] and Stillinger-Weber (SW) [45] potentials have been proved to be the particularly feasible for modeling materials with a diamond cubic structure. To investigate the mechanism of Si in TBN process, the dislocation slip and phase transformation should be observed in MD simulations. Compared to the Tersoff potential, the SW potential not only has enough flexibility to describe a number of different silicon configurations, but also provides the closest match to ab initio dislocation nucleation results in defect-free silicon [46, 47]. Thus, the SW potential function may have more potential to describe the interaction between silicon atoms. Analytical bond order potential (ABOP), Reactive Empirical Bond Order (REBO), and Adaptive Intermolecular Reactive Empirical Bond Order (AIREBO) potentials are a class of potentials which extend Tersoff’s potential function according to the tight-binding approximation and rely on fundamental quantities [48]. The ABOP potential is ideal for the interactions between the atoms of silicon and carbon, which is employed to investigate the material deformation and the removal behavior in the process of SiC. The REBO potential is particularly popular in carbon and carbon nanotubes simulations. The AIREBO model was developed to overcome the deficiencies of REBO potential function providing more accurate approximation than those of REBO terms and also added capabilities of modeling more complex interactions [41]. The inter-molecular and intra-molecular interactions in the polystyrene specimen are described by the well-established AIREBO potential [49].

The majority of MD simulations was performed with the use of Large-scale Atomic/Molecular Massively Parallel Simulator (LAMMPS) [50]. Some complex workpiece models like polycrystalline and nanotwined polycrystalline materialscould be carried out with Atomsk [51]. The construction of polymers, including atoms, bonds angle, improper, and their various types are obtained from the data file generated from Material Studio (MS) [52]. Both Open Visualization Tool (OVITO) [53] and visual molecular dynamics (VMD) [54] are useful tools to visual the model or the process of machining.

With the use of MD simulations, various materials models could be established effectively. However, most workpiece models are smaller than 50 nm × 50 nm × 50 nm in size, which may result in the deviation from the real results [40, 55, 56]. Besides, there is still a lack of potential functions which could effectively describe some materials like GaAs and Lu_2_O_3_. Thus, MD models and related potential functions still need to be optimized to describe the simulation process more accurately.

### Processing Configuration

In TBN experiments, the load-controlled mode is usually carried out by applying a constant normal load on the tip. This mode has been demonstrated to be of higher precision, especially conducting nanomachining on inclined or curved surfaces. However, many MD simulation of TBN processes are performed under displacement-controller mode, which may lead to difference between simulation and experiment [18, 57]. Thus, some scholars have performed MD simulation of load-controlled nanoscratching process by directly applying a constant normal load on the probe [20, 58]. In the machining processes, it can be divided into three parts: relaxation stage, penetration stage, and scratching stage. In addition, the tip is not completely perpendicular to the workpiece because of beam bending of the atomic force microscope (AFM), substrate with a tilt angle, as well as roughness of the sample surface. Thus, the effect of the tip tilt should not be neglected. Liu et al. chose tips with different inclination angles to investigate the effect of the tip inclination on the machining outcomes [59]. In addition, the tip wear is a key factor for the machining process, which has large influence on the machining quality. Nanoscratching with water-layer lubrication may reduce the tip wear to increase the tool life and guarantee the machining quality. However, the machining mechanism affected by the water-layer lubrication is still not well understood. To solve this point, Ren et al. used MD simulation method to investigate the effects of water-layer lubrication on the machining results with a monocrystalline copper [60].

**Fig. 2:**
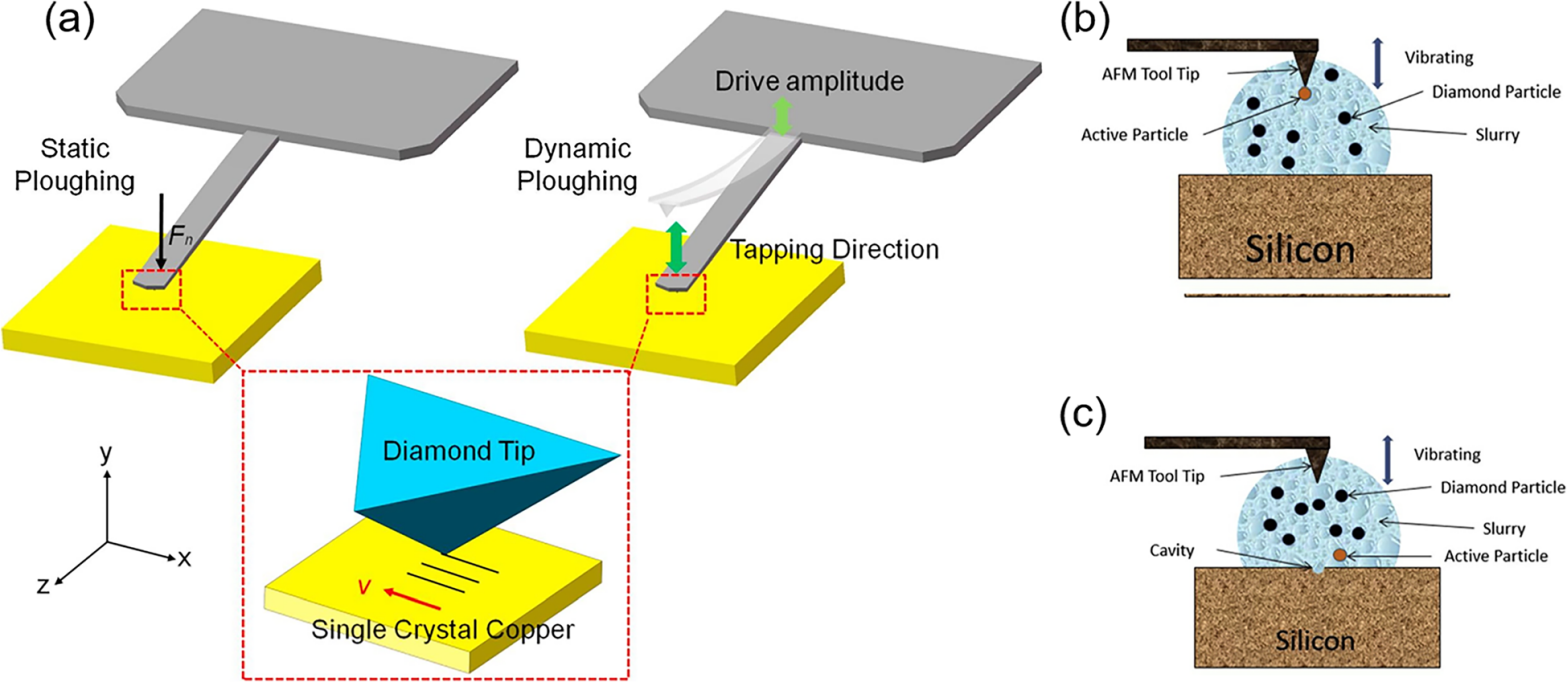
**a** The schematics of the static and dynamic ploughing lithography on single crystal copper [19]. **b** AFM tool tip striking a diamond particle. **c** diamond particle striking the workpiece [61]

Single scratch process has been proved to be an easy method to fabricate nanogroove, but it has the limitation on the dimension of the groove. The multi-pass scratching method was presented to enlarge the size of nanogroove [62]. Geng et al. have studied the difference between single-pass approach and multi-pass approach with MD simulation and experimental results [20]. Both single-pass and multi-pass approaches can be considered as static ploughing lithography, which are conducted with the contact mode of AFM system. However, the static plough lithography may induce non-negligible tip wear when carrying out long-distance scratching process. Tapping mode-based machining technique is named as dynamics ploughing lithography, which have the potential to reduce the tip wear. Figure 2 shows the schematics of the static and dynamic ploughing lithography on single crystal copper, respectively. For the static ploughing, the diamond tip firstly fed downwards and then started ploughing along the negative direction of the *x* axis. After the ploughing was finished, the tip was withdrawn upwards to the original vertical position. For the dynamic ploughing process, the diamond tip moved along a sinusoidal curve with a pointed peak-valley amplitude and period [19]. Based on the principle of tapping mode-based machining technique, Sundaram et al. have developed a novel nanomachining method using AFM, which is referred to Vibration-Assisted Nano Impact machining by Loose Abrasives (VANILA). In this method, the AFM is used as a platform and nanoabrasive are injected in the slurry between the silicon workpiece and the vibration of AFM tip. The kinetic energy for the abrasives is generated by the vibration of the AFM tip and consequently results in nanoscale material removal of the sample [61]. Schematic representation of the VANILA process is shown in Fig. 2b, c.

## Analysis of Simulation Result

### Cutting Force Analysis

The cutting force could clearly reflect the material removal process and defect of evolution, which is an important physical parameter to understanding the cutting phenomenon [19, 63, 64]. In addition, the cutting force is closely related to the cutting power and tool life, which could provide guidance in TBN machining process [65]. In the TBN process, the cutting force is mainly evaluated by the force calibration method, but the lateral force could not be obtained by this method [66, 67]. By using molecular dynamics technology, the variation of cutting forces including lateral and tangential forces can be observed in real-time for the whole process [68]. In addition, the average force and resultant force could also be picked up through MD simulation method.

Though the analysis of cutting force variation, the difference of various material structures and the influence of machining parameters on the TBN process can be reflected. Li et al. found higher forces for scratching on polycrystalline Cu compared with the single crystal Cu is attributed to the fact that the anisotropy of the surface grain has little effect between workpiece and tip when the tip goes from one grain to another grain of different crystal orientations, while the stable plastic flow is shown in material removal of single crystal Cu owing to its single orientation system [25]. It is also found that the cutting force for different crystal structures increases with the increment of cutting speed because a higher nanoscratching speed produces more chips [69]. On the contrary, the change of cutting force presents adverse behavior in the scratching process of SiC, because higher speed can generate more amorphous crystal structure atoms, which makes the SiC material more ductile and easier to be removed [70]. Yan et al. investigated the processability of Cu/Ni bilayers using MD simulation method and found the force of Ni-Cu bilayers is the higher compared with Cu, Ni, and bilayers Cu-Ni, since the movement of dislocations was impeded by the interface of bilayers which served as a barrier of propagation [71]. Concerning about amorphous polymers, the machining property was influenced by the scratching velocity in three different aspects as follows [72]: firstly, the larger pile-up height in front of the tip generated at higher velocity leads to a greater tangential force due to more resistance to the tip. Then, higher velocity leads to a higher deformation rate, which may bring about the strain hardening of the material. This would also lead the increment of cutting forces. Finally, with the increment of the velocity, the thermal softness effect allows the workpiece to be machined more easily, reducing the tangential force and normal force. In the machining processes of polymers, the cutting forces increase with the increment of velocity, indicating that the pile-up and strain rate hardening effect play more important roles in determining scratching forces. In addition to the influence of the workpiece materials, tip geometry also plays an important role in machining process [18, 73, 74]. Ren et al. investigated the effect of tip angles on cutting forces with cone shape tip. It is found that forces increase with the increasing semi-apex angles due to growth of the contact area between tip and workpiece materials [21]. Besides, the friction coefficient decreases strongly with semi-apex, while the hardness increases [75].

**Fig. 3:**
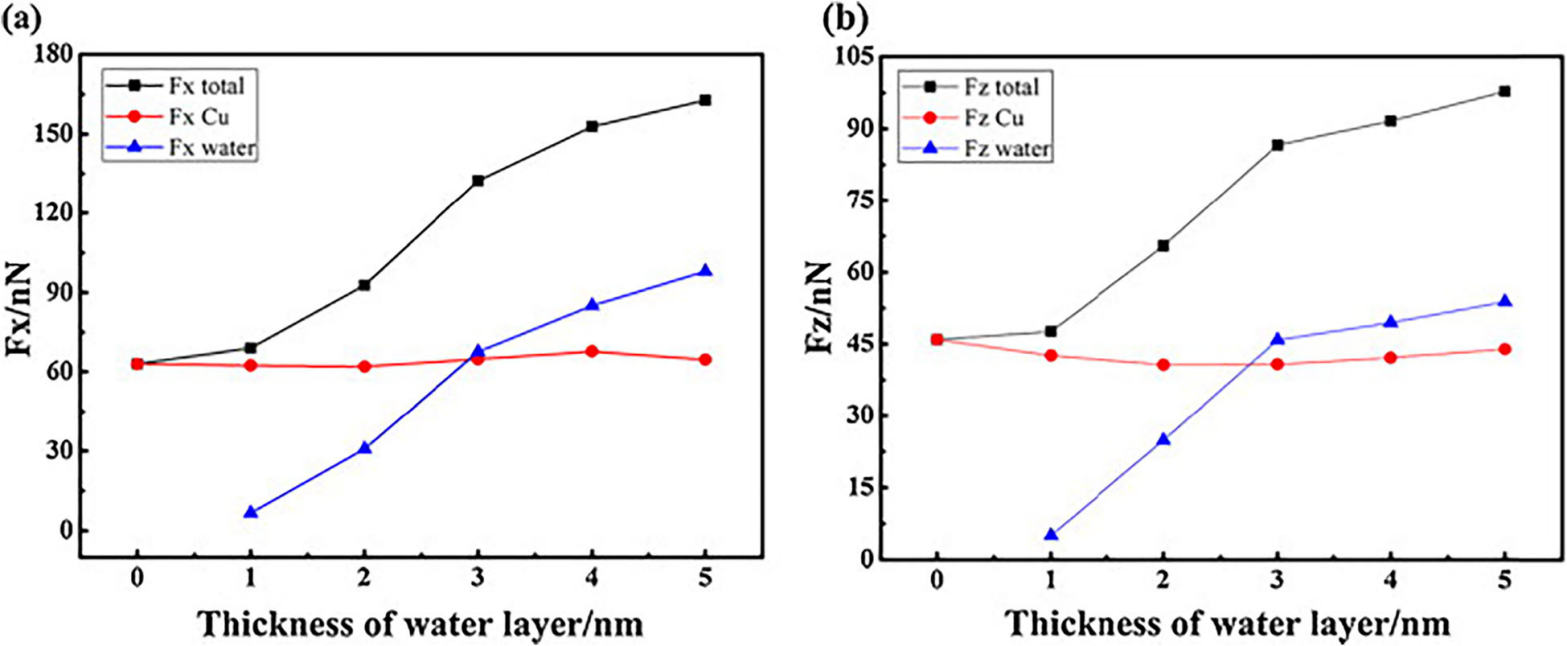
**a** Variation of average scratching forces with change in water layer thickness: **a** tangential forces and **b** normal forces [60]

Some scholars also investigated the variation of cutting force in specific machining conditions. Ren et al. analyzed the correlation between the thickness of the water layer and the scratching forces. Variation of scratching forces with change in water layer thickness is shown in Fig. 3. Unlike the macroscratching process where the water layer mainly plays role of lubrication and reduction of cutting force, the resistance of the water layer is dominant compared with the lubricating effect and the thicker water layer leads to larger total cutting forces [60]. Besides, the effect of tip inclination is also discussed by the analysis of hardness (normal force per atom) and friction coefficient [59]. The results show that the normal hardness is more sensitive in forward or backward direction compared and the effect of tip inclination laterally can be neglected. In addition, the tilt effect on the normal force is the main reason for the change of friction coefficient and the tilt effect on the scratch force is much less than the effect on the normal force.

By comparing the cutting force between static ploughing and dynamic ploughing, the difference of mechanism can be revealed. The cutting force in the dynamic ploughing lithography oscillates drastically with the periodic tapping of the diamond tip, while slight fluctuations are observed in static ploughing. Moreover, the average machining force in dynamic ploughing is smaller than half of that in the static ploughing, indicating less tip wear in the dynamic ploughing lithography [19]. In the dynamic ploughing process, the tip orientation has large influence on the cutting force. Yan et al. combined the groove depth and ploughing force to compare the machining efficiency. The results show that sideface-forward of the tip has the best efficiency as it had the similar ploughing force with that of orientation face-forward of the tip but at the same time more than twice the groove depth [63]. Through the analysis of cutting forces, the mechanism of TBN machining process with various materials and machining conditions could be further explained. Moreover, the comparison of cutting forces in various processing modes could provide guidance for the process optimization of TBN approach.

### Material Removal State Analysis

Material removal state analysis is also an essential method to reveal the mechanism of TBN approach. The removal of the material and topography in machining process are usually observed though scanning electron microscope (SEM) and AFM. However, the groove morphology and removal state can only be detected after processing and the material removal process could not be dynamically understood. Recently, Zhang et al. used linear cutting tools directly connected in SEM to see the chip formation process in material cutting, while this method is complicated and SEM should be modified [76]. Thus, this method is difficult to be generalized. Compared with the experimental method, the MD simulation method can be easily used to explore the material removal mechanism and surface generation in nanoscale, and the machining process can be observed in real time though MD method.

It is known from MD simulation results of TBN process that the deformation states are classified into ploughing state and cutting state. With the movement of the tip, the workpiece material atoms ahead of the tip are squeezed and then accumulate to form the continuous chip during cutting state. Simultaneously, there are also some workpiece material atoms pile up on the left and right sides of the fabricated groove after the passing of the tip [57]. By comparing the ratio of the cutting state to the non-cutting state, we can get whether the cutting state or the ploughing state take up the dominant part when the various tip radius is used to scratch at the different scratching depths [77]. For the past few years, many scholars have further investigated the material properties on the material removal states. For example, single crystalline material shows anisotropy in TBN process, which has large effect on the material removal [20]. Compared with single crystalline materials, grain boundaries have an important effect on the mechanical properties of polycrystalline materials. Gao et al. found that grain orientation rather than grain size is also dominant in determining the profile of the pile-up [78]. Moreover, the tip geometry also has strong effect on the material removal. Three types of the tip (conical, triangular pyramid and hemispherical tip) were selected to reveal the effect of the tip geometry on the material removal state. For the conical tip, there is a clear dependence on the half apex angle. With a bigger half apex angle of tip leads to more chip volume and improves the smoothness of scratching surface, but requires higher scratching force, generates larger friction coefficient and higher temperature in workpiece, and increases subsurface damage [40]. Alhafez also investigated the effect of the half apex angle on the formation of pile-up [75]. It is found that when scratching with small half apex angle of the tip, the pile-up mostly accumulates in forward direction, while the lateral pile-up dominates for the machining condition of the large half apex angle of the tip. Some studies focused on the triangular pyramidal tip which is consistent with the real geometry of the tip in AFM-based machining process [19, 20, 63, 79]. Three machining directions were mainly compared, which are edge-forward, face-forward, and sideface-forward. In the machining process of SiC, the material removal state can be simply controlled by adjusting the scratching direction of the tool. The edge-forward scratching direction can provide a more stable process, which can result in better size accuracy and consistency of the obtained grooves [79]. However, the material removal state of polymers is more sensitive to temperature compared with metal or semiconductor materials. During the scratching process, the local temperature in the scratching zone is higher than glass transition temperature, which indicates that the workpiece in the scratching zone can be removed in a ductile manner [72]. Zhan et al. investigated the microscopic friction mechanisms of amorphous polystyrene. They found that the flexibility of the molecular chains goes up with the increasing of temperature, which can enable the molecular chains to more easily curl and return to their original state [56]. Du et al. found that machining velocity has large influence on machined results of polystyrene. The intra-chain change dominates the permanent deformation of polystyrene specimen when machining velocity is small, while the inter-chain sliding is more pronounced when machining velocity is large [80].

**Fig. 4:**
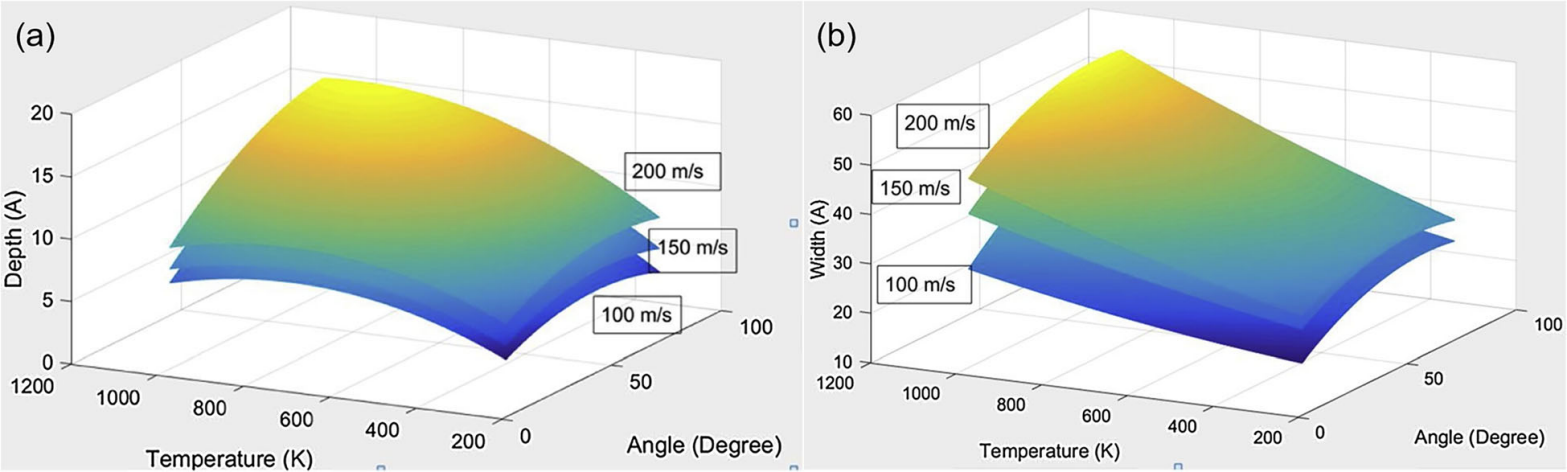
Multiple linear regression plot for **a** depth of the nanocavity (impact speed, impact angle, and operating temperature) and **b** width of the nanocavity (impact speed, impact angle, and operating temperature) [61]

As can be seen from the above discussion, most of studies mainly focused on the dry condition instead of fluids. However, atoms accumulated ahead of the tool are reduced and the burrs along the grooves are not obvious with high thickness of the water layer and the surface roughness decreases visibly and results in a relatively smooth surface. It can be explained that the water layer acts as the lubricant to reduce the sticking region between the tool and the freshly formed chip surface and reduces drag force exhibited at the chip-tool face boundary. The direct consequence is that the surface quality is improved greatly. With the increasing of the thickness of the water layer, the surface quality can be improved gradually [60]. In some novel machining approaches, Shockly et al. investigated the effects of parameters (impact speed, impact angle, and operating temperature) in the vibration-assisted nanoimpact machining on the formation of nanocavity and found that the operating parameters have substantial influence on the depth and width of the generated nanocavities as shown in Fig. 4 [61]. Xiao et al. found the depth and width of groove in the dynamic ploughing process are smaller than those in the static ploughing process, which means that nanostructures with small features could be fabricated through dynamic ploughing lithography. Besides, the dimension grooves could be controlled by the drive amplitude ratio in the dynamic ploughing process, and this demonstrates that the fabrication of the grooves could also be controllable [19].

During the material removal process, wear behavior exists on the tip. In most of the previous studies, the tip is defined as a rigid body, which means tool wear phenomenon could not be observed directly [13]. Many scholars investigated tool wear by studying material removal state, stress, and temperature distribution [70, 77]. In order to reflect the real wear phenomenon of tip during the machining process, Meng et al. set the tip as deformable body and found wear behavior of the tip during the machining process [81]. The results show that the form of diamond abrasive wear is mainly adhesive wear at the beginning stage and atom-by-atom wear at the processing stability stage and the amount of atom-by-atom attribution wear of abrasive is less affected by cutting speed [81]. It is noticeable that there are relatively few reports on wear behavior of the tip. Hopefully, using the deformable body of the tip is expected to further promote the investigation of tip wear behavior during the nanomachining process.

Material removal state analysis provides an effective support for real-time monitoring of machining process. However, due to the limitation of the length scale as mentioned above, the morphology of the machined surface and materials removal process could only be analyzed by qualitative comparison. It is difficult to predict the accurate processing results.

### Defect Evolution Process Analysis

To obtain the information about the defects generated during the scratching process, TEM is usually employed after the FIB sample preparation techniques, which has several disadvantages, such as relatively complicated operation, high cost, and strong material dependence.

**Fig. 5:**
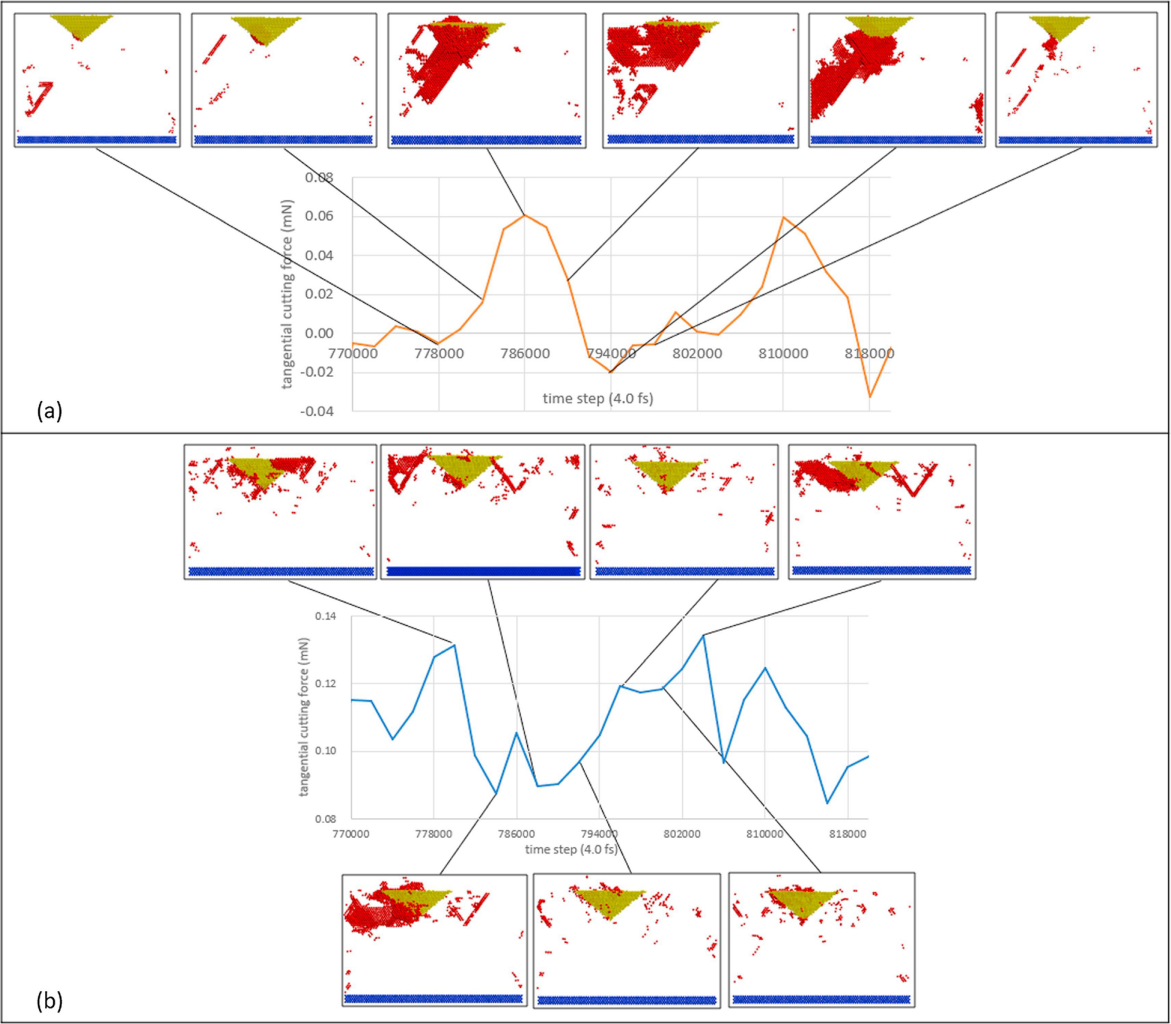
Slipping processes in **a** dynamic ploughing and **b** static ploughing [19]

The MD simulation method can obtain the defects generated beneath the sample surface easily. Moreover, the defect evolution process during the scratching could also be observed by using the MD simulation approach, which could not be obtained by experimental method. Many available algorithms to extract defect types were presented, including common neither analysis (CNA) [82], centro-symmetry parameter (CSP) [83], slip vector analysis [84], Ackland-Jones analysis [85], etc. However, these methods are not suitable for tracing the propagation of dislocations, especially when a large number of dislocations are generated by tip scratching along certain direction, and we can no longer investigate what is going on inside the specimen using these methods [86]. Chen et al. presented slipping analysis for visualizing the atomic slipping process for material deformation, which could filter out those atoms that have slipped relative to its neighbor atoms during a specified period of time in the condition with large numbers of atoms [86]. By using this method, Xiao et al. investigated the slipping process during dynamic and static ploughing lithography [19]. The relationship between the cutting force and slipping process is shown in Fig. 5. It could be seen that for dynamic ploughing, the sample material mainly flows downwards and sidewards, whereas no obvious downwards material flow is observed in static ploughing process. In addition, the propagation of the dislocation is dependent on the orientation of the tip, leading to the various morphologies of the grooves. Dislocation extraction algorithm (DXA) is also a useful dislocation analysis tool to identify the lattice dislocation and to determine their Burger vector [87]. By using this method, Gao et al. investigated the behavior of the nanoscratching of iron. They found that a distinct reorganization of the dislocation network. At the beginning, the plastic zone grows linearly with the scratching length along the path. Then, the dislocation density decreases rapidly after some length dislocation reactions. Plastic activity then is concentrated only on the scratch front. Only few dislocations remain in the middle of the scratch. Vacancies in this zone are created by dislocation reactions. It is also found that point defects vacancies generated by dislocation reactions and deformation twining [88].

For the polycrystalline materials, the deformation mechanism was mediated by dislocation nucleation within grain interior as well as grain boundary dislocations in polycrystalline material [43]. While the dislocation propagation is associated with the formation of nanovoids and interstitial clusters in the case of larger grain size, and the formation of twins at the grain boundary was dominating for smaller clusters. This behavior was attributed to the transition of dislocation movement from smooth (larger grain) to rough (smaller grain) during scratching process. Li further concluded the difference of material deformation mechanism about single crystal Cu, polycrystalline Cu, and NT polycrystalline Cu. The results show that the plastic deformation is mainly affected by the interaction between dislocations during scratching process in single crystal Cu; while for polycrystalline Cu both dislocations and GB dominate the plastic deformation; and the plastic deformation is controlled by the interactions of the dislocation, grain boundary (GB), and twin boundary (TB) accompanied with twinning/detwinning [25].

Furthermore, many studies focused on the MD simulation of the TBN process on the semiconductor materials, such as silicon, silicon carbide, gallium arsenide, and aluminum nitride. To investigate these brittle materials, phase transformation is also an important deformation mode in addition to dislocation slip [89]. The interaction between dislocation and phase transformation varies with the crystal orientation. The results indicate that prior to the “Pop-In” event, Si (010) undergoes inelastic deformation accompanied by the phase transformation from the Si-I to the Si-III/ Si-XII, which is not occurred in Si (110) and Si (111). While, the phase transformation from the Si-I to the bct-5 is the dominant mechanism of incipient plasticity for each crystallographic orientation, and dislocation nucleation is also an operating deformation mode in the elastic-plastic transition of Si (010). Dai et al. investigated the subsurface damage mechanism on single crystal silicon during TBN process. It is found that the evolution of crystalline phases is consistent with the distribution of hydrostatic stress and temperature [40]. SiC is also a kind of important semiconductor material, which has the similar property as silicon. The SiC material removal process is achieved through the phase transfer from zinc blended to amorphous structure with few hexagonal diamond structures. Higher scratching speed generates more amorphous structure atoms, fewer hexagonal diamond atoms, and fewer dislocation atoms due to larger impaction and less rearrangement time [90]. While Meng et al. found when the phase transition is not the dominant deformation mechanism, the Schmidt coefficient method can effectively predict the sliding motion of 3C-SiC during the TBN process (elastic sliding motion and dislocation slip motion) [79]. Moreover, Meng et al. further studied on the strain rate and heat effect on the removal mechanism of SiC. They found that the strain rate effect and the thermal softening effect directly affect the material removal amount and form of the subsurface damage (SSD). The influence of the thermal softening effect on the stress in the processing region under the condition of high strain rate exceeds that of the decrease in the growth rate of the dislocation generation speed. The polycrystalline SiC removal process is dominated by the amorphous phase transition. Furthermore, several hexagonal diamond structure atoms and dislocations are found in the GBs during the scratching. Higher scratching speed and larger depth of cut promotes more atoms to transfer into the amorphous structure due to larger impaction [81]. Compared with monocrystalline SiC, the microstructure in polycrystalline makes the SiC more soften by generating less normal scratching force and amorphous structure phase transition and thinner plastic deformation induced SSD [91]. Dislocation propagation and phase transition analysis could explain the mechanism in machining process. However, most researches focused on single crystalline materials and the materials with complex structure are rarely reported, which should be further studied.

## Future Research Directions and Challenges

At present, the research on the TBN process through MD simulation is widely reported. However, there are still some limitations to be considered. Thus, future directions are discussed in this review.

1. With the development of TBN methods, some novel technologies have been proposed in this field. For example, AFM tip-based nanomilling process has a broad prospect due to its great machining performance and size control properties [92, 93]. However, the mechanism of nanomilling has not been fully understood due to limitation of the detection equipment. With the use of MD simulation, it is hopeful that the variation of the cutting force, the dynamics change of defects, and the removal state of workpiece materials during rotating process of the tip. In addition, sample vibration-assisted nanoscratching method has not been reported yet. MD simulation approach could provide meaningful guidance in the early stage.
2. Due to the limitation of the length and time scales, MD methods still could not fully describe the experimental process quantitatively. In some studies combining experiments and MD simulations, MD simulation approach could only qualitatively explain the experimental phenomena [19, 20, 94]. In particular, for some time-dependent materials such as amorphous polymers, the velocity of the probe has a significant impact on the removal state of polymers. Thus, in order to accurately predict the experimental process and quantitative analyze the experimental results, the improvement of algorithm and computing capability is indispensable.
3. Many nanostructures have been achieved on polymer materials using the TBN method [95–97]. In particular, the mechanical machining process of polymer materials based on TBN method keeps the normal load constant so as to guarantee the accuracy of the machined nanostructures [98]. However, the reports of MD simulation of polymer in TBN process are limited until now. Moreover, thermal scanning probe lithography is developing in polymer, which has the potential to improve the machining accuracy of the TBN process. It is necessary to reveal the material removal mechanism of the polymer materials when conducting nanoscale scratching process with the mechanical-thermal effect.

## Conclusion

It is undeniable that MD simulations technology plays an increasingly crucial role in nanomachining process to reveal hitherto unknown phenomena [99]. This review concluded the recent progress in MD simulation of TBN method, and the above contents are summarized as follows:

1. The establishment of MD models of various materials and related potential function were summarized. In particular, the modeling process of NT polycrystalline materials and amorphous polymers were discussed in this section. The accuracy of MD models is of great significant to the subsequent simulation results.
2. The new technologies of TBN methods, including multi-scratching, dynamic ploughing, and VANILA were presented. With the help of MD simulation, the mechanism of these methods could be better understood. In particular, the essential difference between static ploughing and dynamic ploughing was revealed by MD method from the aspects of internal defects, morphology and cutting forces.
3. The analysis of MD simulations in TBN process, including the cutting force, the state of material removal, and defect analysis are also summarized. Besides, some novel analysis methods like slipping analysis are also given. With these methods, the processing mechanism based on TBN approach is reviewed, which shows the materials dependence on the TBN machining process.

## Abbreviations

TBN: Tip-based nanomachining
AFM: Atomic force microscopy
MD: Molecular dynamics
NT: Nanotwined
PE: Polyethylene
EAM: Embedded atom method
SW: Stillinger-Weber
ABOP: Analytical bond order potential
REBO: Reactive Empirical Bond Order
AIREBO: Adaptive Intermolecular Reactive Empirical Bond Order
LAMMPS: Large-scale Atomic/Molecular Massively Parallel Simulator
MS: Material studio
OVITO: Open Visualization Tool
VMD: Visual molecular dynamics
VANILA: Vibration-Assisted Nano Impact machining by Loose Abrasives
SEM: Scanning electron microscope
CNA: Common neighbor analysis
CSP: Centro-symmetry parameter
DXA: Dislocation extraction algorithm
GB: Grain boundary
TB: Twin boundary
SSD: Subsurface damage
